# An anion exchange membrane sensor detects EGFR and its activity state in plasma CD63 extracellular vesicles from patients with glioblastoma

**DOI:** 10.1038/s42003-024-06385-1

**Published:** 2024-06-03

**Authors:** Nalin H. Maniya, Sonu Kumar, Jeffrey L. Franklin, James N. Higginbotham, Andrew M. Scott, Hui K. Gan, Robert J. Coffey, Satyajyoti Senapati, Hsueh-Chia Chang

**Affiliations:** 1 Department of Chemical and Biomolecular Engineering, University of Notre Dame, Notre Dame, IN 46556 USA; 2 Department of Medicine, Vanderbilt University Medical Center, Nashville, TN 37232 USA; 3 Department of Cell and Developmental Biology, Vanderbilt University School of Medicine, Nashville, TN 37232 USA; 4 Tumour Targeting Laboratory, Olivia Newton-John Cancer Research Institute, Melbourne, VIC Australia; 5 Department of Molecular Imaging and Therapy, Austin Health, Melbourne, VIC Australia; 6 School of Cancer Medicine, La Trobe University, Melbourne, VIC Australia; 7 Department of Medicine, University of Melbourne, Melbourne, VIC Australia

**Keywords:** Diagnostic markers, Cancer prevention

## Abstract

We present a quantitative sandwich immunoassay for CD63 Extracellular Vesicles (EVs) and a constituent surface cargo, EGFR and its activity state, that provides a sensitive, selective, fluorophore-free and rapid alternative to current EV-based diagnostic methods. Our sensing design utilizes a charge-gating strategy, with a hydrophilic anion exchange membrane functionalized with capture antibodies and a charged silica nanoparticle reporter functionalized with detection antibodies. With sensitivity and robustness enhancement by the ion-depletion action of the membrane, this hydrophilic design with charged reporters minimizes interference from dispersed proteins, thus enabling direct plasma analysis without the need for EV isolation or sensor blocking. With a LOD of 30 EVs/μL and a high relative sensitivity of 0.01% for targeted proteomic subfractions, our assay enables accurate quantification of the EV marker, CD63, with colocalized EGFR by an operator/sample insensitive universal normalized calibration. We analysed untreated clinical samples of Glioblastoma to demonstrate this new platform. Notably, we target both total and “active” EGFR on EVs; with a monoclonal antibody mAb806 that recognizes a normally hidden epitope on overexpressed or mutant variant III EGFR. Analysis of samples yielded an area-under-the-curve (AUC) value of 0.99 and a low p-value of 0.000033, surpassing the performance of existing assays and markers.

## Abstract (web summary)

Targeting conformally-active EGFR on small extracellular vesicles using a novel Charge-gated Ion Exchange Membrane sensor allows sensitive and non-invasive detection of Glioblastoma in human subjects from untreated plasma.

## Introduction

Liquid biopsy is an emerging non-invasive approach for detecting circulating cancer biomarkers in various body fluids^1^. Extracellular vesicles (EVs), including exosomes and microvesicles, have gained interest as a target for cancer detection^2^. EVs play a role in paracrine cell signalling as nanocarriers for the exchanged RNAs and proteins^3^. They possess the ability to transport highly charged and hydrophilic molecules across the hydrophobic bilayers of cell membranes, and they provide protection to miRNA and mRNA from degrading agents^1^. Furthermore, EVs and their cargo are excreted abundantly and exhibit high stability in various body fluids, such as blood, urine, and saliva^4^. This stability and abundance have motivated the development of diagnostic assays based on EV biomolecular contents.

Here, we focus on one such aspect – the tumour-specific epitope on the CR1 domain of the Epidermal Growth Factor Receptor (EGFR) present on the surface EGFR-amplified cells^5^ and their secreted EVs^6^. Under normal conditions in healthy cells, this epitope is transient and mostly hidden due to a disulfide-bonded loop between amino acids 287 and 302 on EGFR, which creates steric hindrance with the CR1 region^7^. However, in EGFR-amplified cancers, the disruption of this bond in multiple EGFR copies reduces this steric hindrance, making the epitope accessible in the untethered EGFR^7^. A similar exposure of the epitope occurs in the mutant variant III EGFR^8^, where the deletion of amino acids 6-273 exposes the same epitope due to the deletion of L1 and CR1 domain. Therefore, an antibody (mAb 806) specifically targeting this sterically hidden epitope would be ideal for detecting cancer EVs secreted by EGFR-amplified tumour cells^9^, as the same active version of EGFR (aEGFR) is also shared on the tumour-secreted EVs^6^. Other EGFR antibodies such as cetuximab and panitumumab are distinct from mAb 806 as they recognize total wtEGFR (tEGFR)^10^.

However, a key challenge associated with detecting aEGFR using mAb 806 is the relatively high dissociation constant^7^ ${K}_{D} \sim 30{nM}$, while the concentration of aEGFR in plasma is much lower with overall tEGFR concentration < 1–10 pM^11^. Most of the current approaches for examining proteins on EVs, such as Enzyme Linked Immunosorbent Assay (ELISA) and immunoblotting. These methods typically have a limit of detection that is 10–100 times lower than ${K}_{D}$^12,13^ and hence insufficient for robust detection of dispersed aEGFR from lysed EVs. Furthermore, these approaches often require laborious ultracentrifugation and enrichment steps to visualize a band, which becomes even more difficult when working with human plasma due to the presence of high contaminant from non-EV species. Efforts to isolate EV also lead to variation due to high EV loss ( ~ 90%) when high-force ultrafiltration or ultracentrifugation is used to isolate them from other proteins and lipoproteins^14^ leading to a yield bias. Similarly, fluorescent labelling of aEGFR on EVs for flow cytometry or nanoparticle tracking analysis requires EV isolation from plasma to remove unincorporated fluorophores and overcome high labeling interference from dispersed proteins and reactive oxidative species^15^. Autofluorescence from abundant dispersed proteins, such as albumin, is also an issue for any optical assay of raw plasma that involves fluorescent labelling^16,17^.

An alternative approach to quantify aEGFR is to capture EVs with a tetraspanin EV marker (CD63) with high capture affinity and report it with anti-aEGFR at very high reporter concentrations $\gg {K}_{D},$ leading to a sandwich scheme with irreversible antibody association with the *colocalized protein* (aEGFR). However, due to endogenous and handling-induced variations in total EV number, absolute quantification of aEGFR+ EV leads to poor p- statistics. Instead, capturing a fraction of CD-63 EVs with aEGFR colocalized in untreated plasma sample would reduce false positives and negatives due to EV number fluctuations. This normalized colocalization assay requires a large sensor dynamic range because the dynamic range determines the lowest colocalized fraction that can be accurately determined. When captured with tetraspanins and reported with a particular protein of interest, the signal is essentially below the limit of detection if the colocalized fraction is only 1% of the captured EVs in a sensor with a 2log_10_ dynamic range.

Therefore, immuno-sandwich characterization of aEGFR-positive EVs necessitates three key requirements. First and foremost, a high sensitivity detection method is essential to accurately detect the low concentration of aEGFR-EVs or any other EV subfractions in plasma. Secondly, the sensor employed should possess a large dynamic range to enable the reliable determination of colocalized fractions even at low levels. Finally, it is crucial to develop an interference-free approach that eliminates the need for laborious EV isolation procedures. By fulfilling these requirements, the detection and characterization of aEGFR-positive EVs in untreated plasma can facilitate advancements in liquid biopsy-based cancer diagnostics.

Many of the recently proposed immuno-sandwich colocalization assays fail to meet these requirements. Commercial interferometry-based colocalization assays^18^ typically only exhibit one-log dynamic range and hence cannot accurately estimate a colocalization fraction below 10 percent. Commercial nanoflow cytometry and ELISA for EVs use fluorescent reporters and hence require elaborate isolation, blocking and wash steps to remove unbound and non-specifically adsorbed reporters. All these steps can lead to analyte loss and hence diminish the assay sensitivity and dynamic range. Additionally, these manual steps are highly personnel-intensive and operator-sensitive, rendering them insufficiently robust as clinical tools. More recently, literature reports have suggested that electrochemical, plasmonic and total internal reflection fluorescence (TIRF) EV assays yield much better sensitivity than ELISA—10^8^ EVs/ml (fM) for electrochemical sensors^19^ and 10^1^ to 10^4^ EV/ml for TIRF^20^. However, all these new sensors still require optical or redox reporters that can absorb non-specifically. Electrochemical assays also suffers from abundant interfering redox agents in plasma^21–23^. All hence require extensive EV isolation, blocking and wash steps to remove unbound reporters. Signals from all three sensors are also sensitive to the size of the EV (which can vary by 100 nm) and the location of the reporter on the EV, given that their intensity vary greatly over the Debye screening and the plasmonic/evanescence penetration lengths. Not surprisingly, robust normalized quantification and dynamic range are difficult to achieve for these new assays.

We report here the first normalized non-optical colocalization assay for untreated plasma EVs, with sufficient sensitivity (30 EVs/$\mu L$ of sample) and selectivity to quantify the fraction of CD63 EVs with a disease marker in untreated plasma. The sensor is designed to minimize non-specific binding, without blocking, and interference of the reporters—and with proper accommodation for the size of the EVs. We use a charge sensing strategy with a highly charged silica nanoparticle reporter to minimize signals from non-target molecules and EVs, which are typically weakly charged. The dimension of the charged reporter (50 nm) is selected such that only a single reporter can bind with one target EV, due to steric and electrostatic repulsion, such that the charge signal is identical for EVs of different size. The sensor utilizes the long-range ( ~ 1 mm) ion depletion action of an ion exchange membrane^24–26^ to amplify signal transduction by minimizing Debye screening of the reporter (EV size varies by 100 nm and the plasma Debye screening length is less than 1 nm). With depletion, the field of charged reporter can reach the membrane surface and gate the ionic current, independent of its distance to the membrane due to EV size variation. The depletion also removes any interfering agent. Since the reporter signal is only registered after ion-depletion, it can be accurately quantified by a sensitive transition voltage to the “over-limiting” regime due to an electroconvective instability that occurs because of ion depletion^26–28^. We chose CD63 as the immunocapture target because EVs with CD63 are known to be enriched on EVs released by the endosomal pathway ^29^, whereas CD9+ and CD81+ EVs bud from the cell membrane. While it is not clear which class of EVs, or both, would carry tumor aEGFR, we will validate CD63+ EVs as a tumor aEGFR carrier in this study. We plan to study the role CD9+ and CD81+ EVs on EGFR transport in a latter study.

The platform takes advantage of the strong binding affinity of antibodies towards CD63, which is further enhanced by avidity through a combination of high probe density and the presence of multiple copies of CD63 on the surface of EVs. This optimized capture strategy allows for high-affinity capture of a large number of EVs, maximizing the sensitivity of our assay. Unlike ELISA and immunoblotting, which require over 10^9^ reporters to detect a signal, our platform achieves high sensitivity with only 10–1000 silica reporters. Additionally, the small dimension of our sensor ( < 1 mm) offers the advantage of shorter incubation times for signal registration, especially at very low concentrations^26,28^. Traditional surface assays with larger sensors often suffer from transport limitations and necessitate a larger number of reporters, resulting in prolonged incubation times before any signal can be detected. In contrast, our platform’s lower reporter requirement allows for faster signal registration, typically within a timeframe of approximately 30 min to 1 h. This expedited process enables efficient signal detection even at low concentrations. We have also demonstrated that, after the diffusion time of 30 min for our small sensors, the total captured analyte does not change much, as diffusive depletion of the analyte is confined to a small micro-liter neighbourhood of the sensor^28^.

Once captured, the EVs are exposed to a high abundance of silica reporters that target either aEGFR, tEGFR (total EGFR) or CD63. This abundance of reporters ensures that all available binding sites on the captured EVs with our target protein are engaged, leading to rapid irreversible reporter binding within minutes. High reporter concentration overcomes low affinity or low concentration of target proteins on the EVs. Moreover, with the high probe density enabled by dense active sites on the membrane, the sensor can accommodate approximately 10^7^ silica reporters until saturation is reached^28^. This allows for a wide dynamic range of detection, spanning four to five logs. The signal increases proportionally with the logarithmic concentration of EVs, ranging from 100 to 10^7^ silica reporters. Therefore, our platform can accurately detect and quantify EV subfraction concentrations, even at levels as low as 0.01% experimentally. Finally, we also use the large drag force (several orders higher than on molecular analytes) on EVs and the reporter nanoparticle^30,31^ to improve specificity with an optimized wash protocol. The engineered high sensitivity and selectivity of our sensor allow precise analysis of EV subpopulations within complex biological samples. By reliably detecting and quantifying EVs at low concentrations, our platform can detect rare subfractions of EVs, which can have great implications in various fields including cancer research, biomarker discovery, and disease monitoring.

We validate this new AEM EV immunoassay by estimating the fraction of CD63 EVs with aEGFR in a small pilot cohort. We confirm that, due to irreversible association with the abundant reporters, a universal calibration curve exists that allows a very simple estimate of this fraction from the signals with two different reporters for two different colocalized proteins. We also validate the universality of this normalized colocalization assay with an HDL assay for colocalized ApoA1 and PON1. Finally, we analyze glioblastoma clinical samples and assess the total (tEGFR) and aEGFR fractions in CD63 EVs. With samples from both glioblastoma patients and healthy individuals, we also analyze the scatter of the data and arrive at a p-value of 0.000033 for aEGFR-CD63 EVs and 0.01 for tEGFR-CD63 EVs, each with an AUC of 0.99 and 0.755 respectively, both significantly better than any single-marker EV diagnostic assay with untreated plasma. The AEM sensor has been already verified for robust multiplexed quantification of RNA, DNA, proteins, and lipoproteins in our earlier work^28,31–33^. However, it is particularly relevant to EV diagnostics due to the larger dimension and size variation of EVs. Additionally, we report here its fully automated prototype with near turn-key functionality.

## Results and Discussion

### Sensing platform

**Fig. 1:**
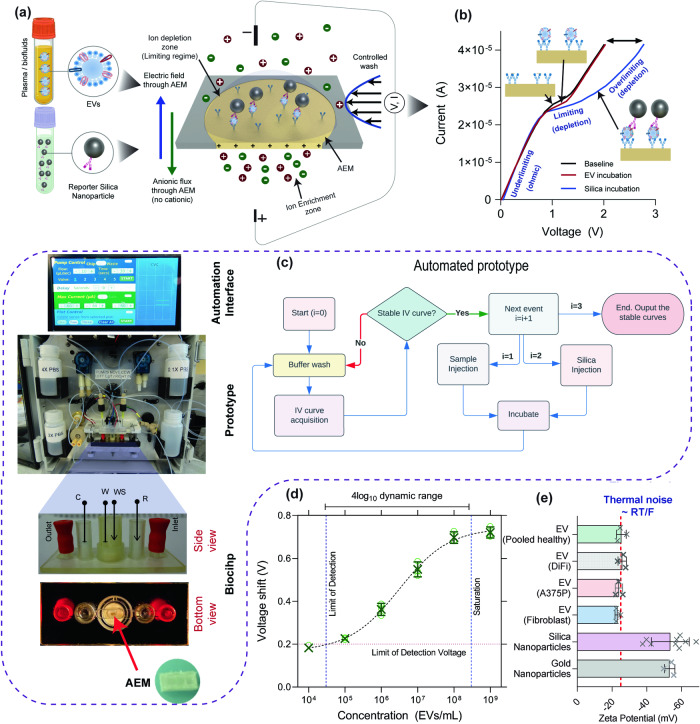
Sensing method utilizes highly negative zeta potential of silica reporters to produce a signal. **a** General schematic and overall workflow of our platform. First, samples containing EVs are incubated, washed, incubated with silica reporters, and washed again. The platform consists of an anion exchange membrane that only allows counter ions to pass through and exhibits three distinct regimes in the current-voltage response. **b** Current-voltage response of an Anion Exchange Membrane: EV does not produce any shift in the overlimiting region, while silica reporters do produce a shift after forming a sandwich due to their highly negative charge. **c** Automation algorithm of our platform with an automation interface, prototype, and biochip showing the housing of AEM. **d** Voltage shift for anti-CD63 capture and anti-CD63 reporter (both monoclonal and of the same clone) that target a specific epitope of CD63 – binding only to species that contain at least two unique copies of CD63 at different concentration of sEVs measured in triplicates **e** Highly negative zeta potential of silica particles compared to EVs from two cell cultures and pooled healthy plasma (all done in triplicates except silica where there are nine replicates). All the error bars are one standard deviation.

The anion exchange membranes (AEM) display characteristic non-linear ion current-voltage (I-V) characteristics due to the ion-depletion action on one surface of the membrane which has been functionalized with the capture antibody ^25,27^. The overall architecture of our platform is shown in Fig. 1a. Schematic I-V curves are shown in Fig. 1b, with a linear Ohmic region at low voltage, followed by a high resistance limiting current regime beyond a critical voltage and then a third overlimiting current regime at an even higher transition voltage, with a differential resistance comparable to the Ohmic region. The automated prototype and algorithm is shown in Fig. 1c. The ion-depletion action is highest at the overlimiting current transition voltage, and it is this transition voltage that is sensitive to the presence of charges on the membrane, and hence registers charge introduced by our capture antibody – EV – reporter antibody – silica nanoparticle complex. The ion-depletion action also ensures the measurements are always made at the same low ionic strength and neutral pH, independent of the sample ionic strength and pH. It hence introduces a universal deionized sensing buffer without extraction and buffer exchange. This makes the sensor ideal for heterogeneous plasma or serum sample analysis as the sensor is insensitive to weakly or non-charged molecules like albumin. Even though these molecules non-specifically adsorb onto the sensor surface, the sensor does not register any signal as the sensor is only sensitive to charge. We hence eliminate the need to block the surface of the membrane sensor or the silica reporter. Of note, excessive fouling by uncharged non-targets will obviously still interfere with the sensitivity and so high probe density with large dynamic range is also required. The sensor measurements are performed at a constant current and the quantification signal is the voltage shift at that current.

It is apparent from Fig. 1b that when EVs bind to the anti-CD63 antibody on the sensor, a voltage shift is not observed. This is because EVs are weakly charged, with a Zeta potential of about −20 mV (Fig. 1e) under low ionic concentrations (near DI conditions). However, as the silica nanoparticles functionalized with a reporter antibody are highly charged^34^ (Zeta potential ~ −50 mV), resulting in a shift when it binds to the EVs on the sensor (Fig. 1d, e) which can be compared against varying bulk concentration to establish a one-to-one correspondence between bulk EV concentration and voltage shift (discussed in detail later). We note that the Zeta potential scales as the logarithm of the charge density, but the total charge is the charge density multiplied by the surface area. Thus, we expect the reporter to introduce at least a few orders of magnitude more charge per EV. Additionally, the wash is done for 10–100 s with PBS which is the typical $1/{k}_{{off}}$ of non-specifically bound non-targets, while the shear force also removes non-specific large targets (such as non-target EVs and aggregates) thus using shear allowing us to greatly improve our selectivity.

### Detection of extracellular vesicles

EV detection was performed in a sandwich scheme using a capture antibody attached to the AEM sensor (Supplementary Fig. 1) and a reporter antibody attached to silica particles. We target the same specific epitope on CD63 using both capture and reporter antibodies due to the high abundance of tetraspanin CD63 marker on EVs secreted by most cell types^2,35^. The concentration of reporter antibodies was maintained at 100 nM, to ensure it is higher than ${K}_{D}$ of high-affinity antibodies. This means that a conservative ${k}_{{on}} \sim {10}^{5}{M}^{-1}{s}^{-1}$, irreversible reporter association is reached within an incubation time of ${({k}_{{on}}{C}_{{reporter}})}^{-1} \sim 100s$. Moreover, the attachment of antibodies to the AEM sensor was confirmed by using Alexa fluor 488 labeled antibodies. The bright green fluorescence observed from the sensor surface (Supplementary Fig. 2a) indicates the successful attachment of the antibodies to the surface. Further, the labeled reporter antibody was used to confirm the attachment of antibodies to the silica particles (Supplementary Fig. 2b). The presence of fluorescence only in the pelleted silica particles shows the efficient functionalization of the antibodies on the silica reporters.

To establish the one-to-one correspondence between the voltage shift observed in the overlimiting regime and the bulk EV concentration we use isolated EVs from DiFi media at increasing concentrations from ${10}^{4}-{10}^{9}$ EVs/mL (Supplementary Fig. 3); in these studies, only one 50 nm reporter can associate with a single EV due to steric and electrostatic repulsion. First, the EV sample was incubated over the biochip for 20 min and then incubated with the reporter for 20 min. After each incubation step, there is a wash step. As seen from Fig. 1b, the IV curves remain the same as the baseline curve with almost no voltage shift after EV binding. The binding of reporter particles caused the drastic voltage shift in the over-limiting region as expected from their zeta potentials. Here, the under-limiting and limiting regions remained constant which can be used as internal control for each sensor response. The blank is obtained by incubating PBS (with no EVs) followed by a silica reporter and is used to determine the limit of detection (LOD) voltage ~0.2 V. The LOD of the AEM sensor was 30000 EVs/mL (quantified by NTA) which is more than 1000-fold lower than the conventional ELISA method (Supplementary Fig. 4) and achieves a four-log dynamic range Figure 1d^36^.

### Specificity of the AEM sensor

**Fig. 2:**
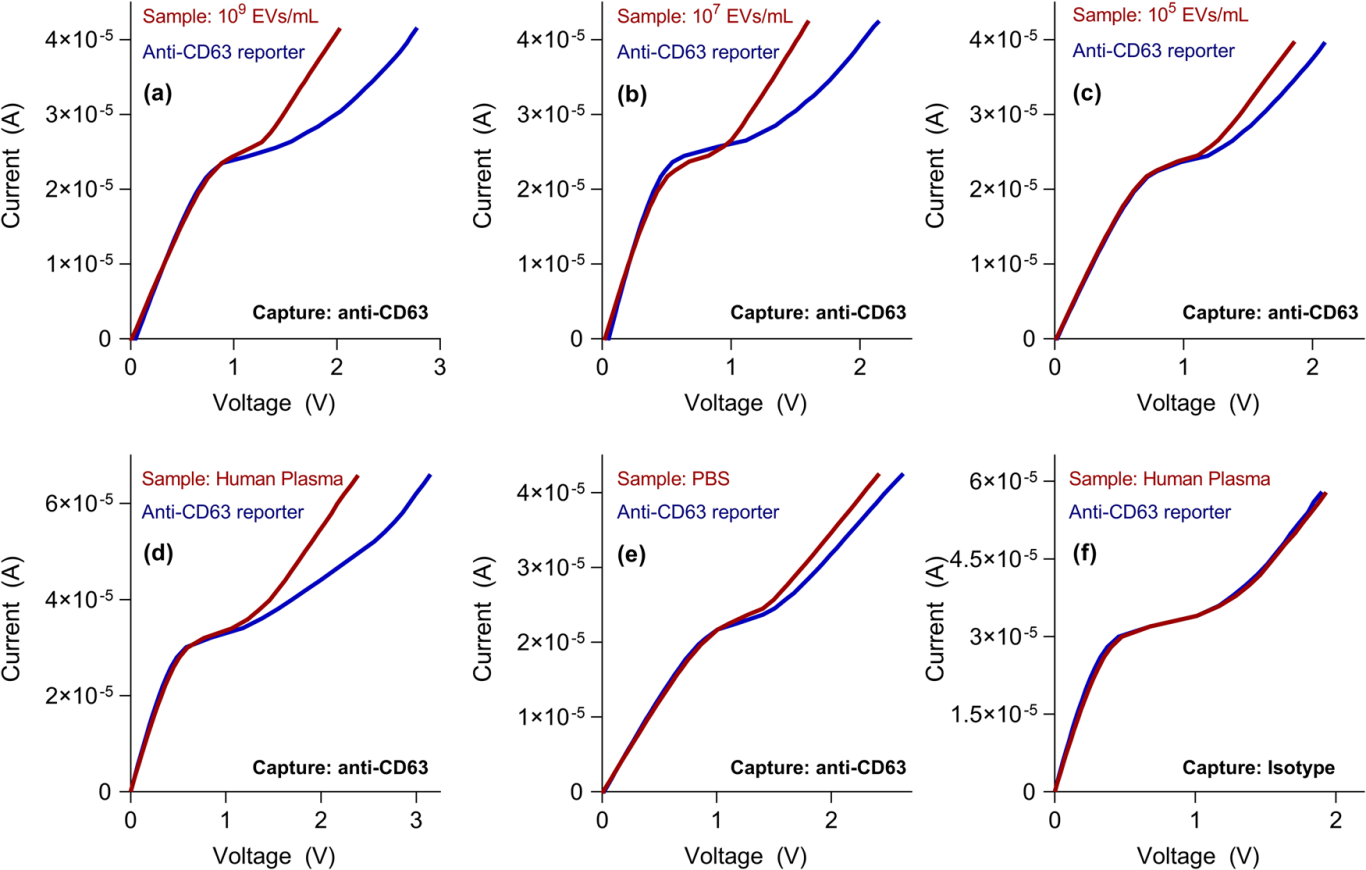
Current-voltage curves for several positive and negative control experiments conducted in our study. **a**–**c** Show the voltage shift with known quantities of EV with anti-CD63 capture and reporter. **d**, **e** Use human plasma and PBS respectively as positive and negative controls to show a small shift produced by the EV-free PBS sample. (**f**) Shift produced by human plasma is negligible with isotype control capture antibody. EVs were isolated from a DiFi cell conditioned medium. Each curve measured for one biological replicate each.

The specificity study of the biosensor is important to make sure that the measured signal is indeed from the specific interaction of antibodies with EVs and not from the non-target interactions between antibodies and interfering molecules from the sample^2^. Therefore, several control experiments were performed to address the specificity. Firstly, as control experiments, the anti-CD63 antibody functionalized sensor was incubated with the EV sample and then with silica reporter particles. As shown in Fig. 2a–c, a large voltage shift was observed from the target sample, despite the low EV concentrations. A large shift was obtained with human plasma with anti-CD63 capture (Fig. 2d), while plain PBS (Fig. 2e) and isotype capture (Fig. 2f) produced relatively low shifts, showing the specificity of our platform obtained from the controlled wash.

### Obtaining Other Subfractions of EVs

**Fig. 3:**
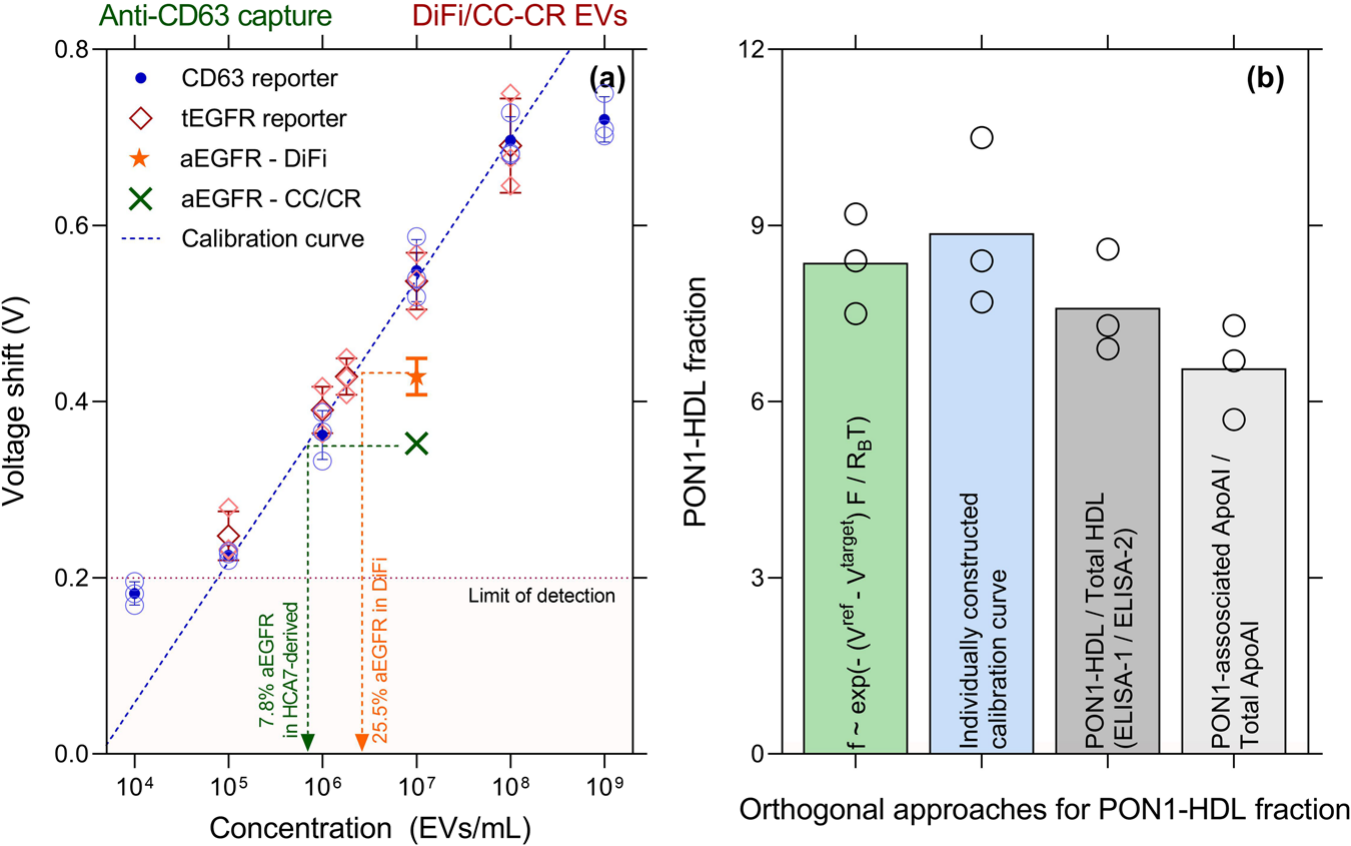
Accuracy of calibration curve in characterization subfractions using our sensor compared to orthogonal methods. **a** Voltage response with anti-CD63 capture and other reporters measured in triplicates. Anti-CD63 reporters and anti-tEGFR reporters give similar responses as they are both present on a vast majority of EVs and because our platform is independent of the affinity of reporter antibody due to the high abundance of reporters. A universal linear calibration between voltage shift and log concentration, corresponding to the linear region of Fig. 1d, is used to estimate the fraction. Each point measured in triplicates. **b** Four orthogonal approaches using untreated plasma demonstrates that the calibration-free universal correlation for the colocalization fraction $f=\exp (-\left({V}^{{ref}}-{V}^{{target}}\right)F/2{R}_{B}T)$, gives a similar estimate as an independently constructed calibration plot (green bar) and two other orthogonal approaches with ELISA-1 and ELISA-2 (grey bar) defined in our previous work^26^. (F is the Faraday constant, T the temperature, and R_B_ the Boltzmann constant from the Boltzmann theory of ion distribution^37^, *V*^*ref*^ and *V*^*target*^ are the voltage shift signal with CD63 and EGFR silica reporters.) Also shown, using a PON1 pulldown and quantifying the ubiquitous protein ApoA1 on the pulled-down volume to normalize against the total ApoA1 (blue bar). HDL was used to verify this universality due to its high concentration and ease of validation using orthogonal methods. Same biological replicate was used across all the methods in (**b**). Error bars are one standard deviation.

Our calibration curves are highly reproducible due to the electrokinetic nature of the signal, eliminating the need for frequent recalibration. A key feature of our platform is the consistent slope observed in the voltage shift versus the logarithm of the (molecular or EV) analyte concentration (slope $\sim 2{R}_{B}T/F$), which holds true across various analytes tested, including proteins and lipoproteins. This universality is related to the logarithm dependence of the Zeta potential to surface charge^26,37^. With our small sensors, we find this calibration curve to be incubation time independent^28,31^. With small molecules, we do find antibody affinity sensitivity such that the standard curve in the semi-log plot of voltage shift versus concentration translates with different antibody-protein pairs while retaining its slope. However, because of high reporter concentration, irreversible reporter association becomes affinity independent. This would produce a universal calibration curve for all target proteins on the CD63-EVs that are reported by the silica particle. To demonstrate this universality, we use antibodies for tEGFR as a reporter which is known to be present in the vast majority of DiFi-derived EVs showing the voltage response remains the same and coincides with the CD63 calibration plot (Fig. 3a)^6^.

**Fig. 4:**
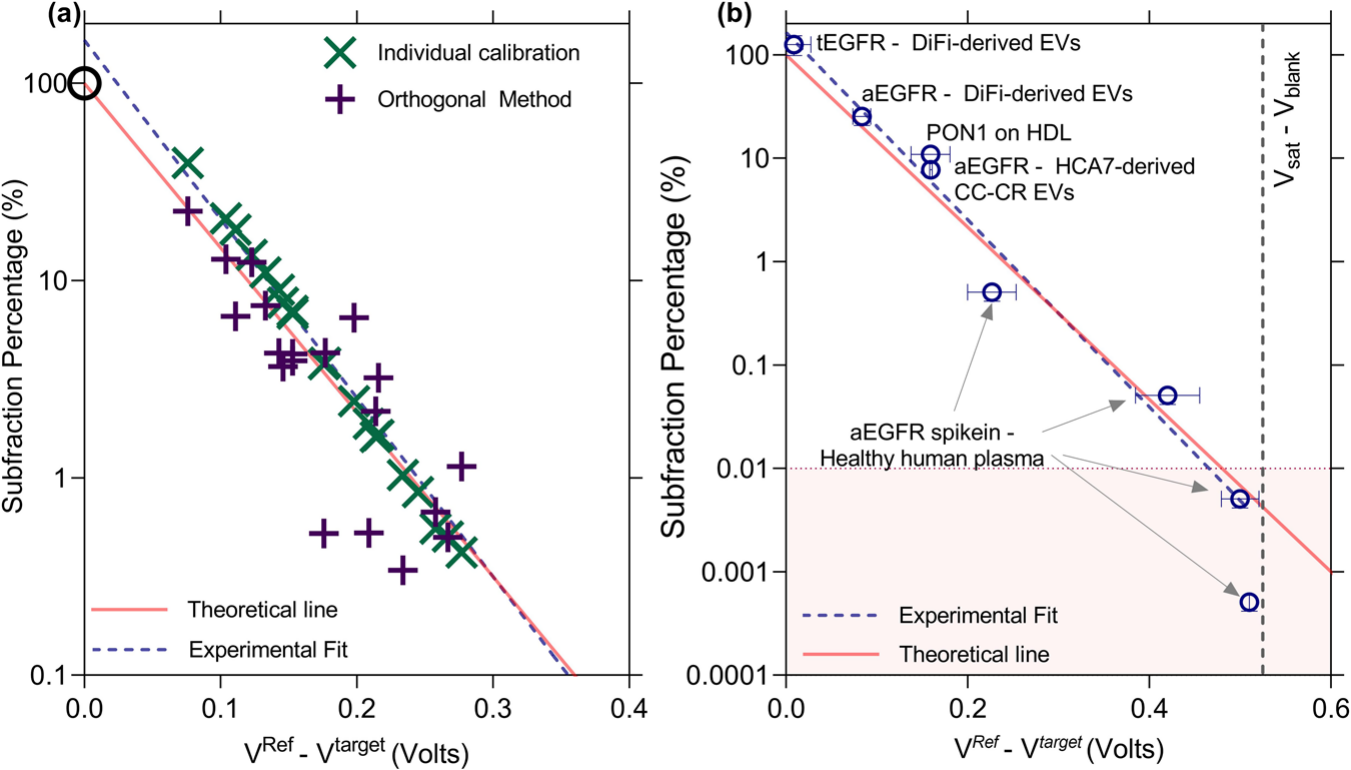
Universality of scaled calibration curve to characterize sEV subfractions. **a** Subfraction percentage estimated from individual calibration curves for PON1-HDL and PON1-free HDL as well as orthogonal method (ELISA-1/ELISA-2) plotted against the difference of ${V}^{{ref}}$ (PON1-free HDL) and ${V}^{{target}}$ (PON1-HDL) using 20 independent human untreated plasma samples. The theoretical line is the universal colocalization fraction $f=\exp (-\left({V}^{{ref}}-{V}^{{target}}\right)F/{R}_{B}T)$ where $-F/{R}_{B}T$ is the slope. The experimental fit shows a similar slope as that of the theoretical line. **b** aEGFR subfractions determined using anti-CD63 calibration curve (reporter independent as previously shown) and plotted against ${V}^{{ref}}-{V}^{{target}}$ for various capture-reporter antibody combinations and for different cell culture media/plasma. The data again follow the theoretical line to confirm that no calibration is needed. This universality is especially useful when determining non-abundant EV fractions that cannot be calibrated easily due to their unknown concentrations. Single biological replicate for each point. Error bars are one standard error.

The universal calibration curve of our platform provides a straightforward approach for normalizing EV measurements against a reference species, regardless of the specific subset of EVs of interest. For a given reporter, our earlier studies^26^ indicate that ${V}^{{target}}=\frac{2{R}_{B}T}{F}{{{\mathrm{ln}}}}(\alpha x{C}_{s})$ where $\alpha$ is a constant related to the zero potential reference and the valency of the charge, ${C}_{s}$ is the concentration of captured species and $x$ is the fraction of captured species having colocalized proteins of interest (see definitions in the caption of Fig. 3). We can similarly write ${V}^{{ref}}=\frac{2{R}_{B}T}{F}{{{\mathrm{ln}}}}(\alpha y{C}_{s})$ where $y$ is the fraction of captured species having reference colocalized protein of interest where the reference could be a ubiquitous protein such as CD63, CD9, or CD81, or even a cocktail. In that case, the percentage of colocalized proteins compared to a reference protein obeys the universal calibration curve $f=x/y=\exp (-\left({V}^{{ref}}-{V}^{{target}}\right)F/2{R}_{B}T)$. It is important that the capture antibody be shared for both reporters (to eliminate ${C}_{s}$). For example, CD63 capture and CD63 reporter can be used as a reference for EVs that are captured with CD63 and produces a shift of ${V}^{{ref}},$ while a reporter for a protein of interest produces a signal of ${V}^{{target}}$ (after CD63 capture). This also implies that the lowest percentage one can successfully characterize is the inverse of the dynamic range span. Since we have a four-log dynamic range, we are able to reach 0.01% (Fig. 4a, b).

To demonstrate the calibration is universal for all CD63-EVs and is independent of the affinity of the capture or reporter antibodies, we use high-density lipoproteins as our model system as they are simpler to characterize using less sensitive orthogonal methods and are in high abundance to use methods such as modified ELISA^28^. In the first approach, using anti-ApoA1 as capture, we obtain two voltage shifts corresponding to the target (anti-PON1 reporter) and reference (anti-ApoA1 reporter). Using the theoretical estimate $f$, we obtain similar values compared to other methods – including constructing individual calibration plots (Fig. 3b), yielding that about eight percent of all HDL from healthy donors contains PON1. The two orthogonal methods yield values that are very close to what we obtained using our method (Fig. 3b). The first orthogonal method, ELISA-1/ELISA-2, is a modified version of ELISA previously used in our work^28^. The second orthogonal method uses the primary protein on HDL (ApoA1) in the immunoisolation and as a reference, with a corresponding voltage shift *V*^*ref*^, and the target PON1 with a voltage shift of *V*^*target*^ to obtain the fraction of ApoA1-HDL that displays PON1. The measured PON1-HDL fraction obeys the same relationship $f=\exp (-\left({V}^{{ref}}-{V}^{{target}}\right)F/2{R}_{B}T)$ as EVs. This colocalization calibration should hence be valid for any nanocarrier, EV, HDL, LDL, etc. Additionally, we compare PON1-HDL (target) and PON1-free HDL (reference) for 20 human participants, and we compare the subfraction from individually constructed calibration plots as well as orthogonal methods (ELISA-1 and ELISA-2 in our previous published work) and observe that the same universal curve for EVs also applies to PON1 + HDL (Fig. 4a)^28^. We note the larger scatter of the orthogonal methods at low concentrations. These methods are prone to many errors even for simpler lipoproteins since lipid peroxides interfere in the enzymatic reaction (this is described in detail in our previous work^28^).

Using the reporter in high abundance to accommodate low-abundance target proteins, we successfully characterized aEGFR (untethered EGFR and EGFRviii) and determined their relative fractions using CD63 calibration plot as well as the theoretical estimate $f$, as illustrated in Fig. 4b. We are able to collapse all colocalization data for tEGFR-CD63, aEGFR-CD63 and PON1-ApoA1 from plasma and cell culture media of DiFi and HCA7-derived CC-CR cell lines using the same universal calibration curve. Additionally, to investigate the minimum detectable subfraction colocalization directly in raw plasma, we conducted spike-in experiments using varying quantities of DiFi-media derived extracellular vesicles (EVs) with known aEGFR fractions. These EVs were introduced into healthy pooled human plasma, which initially had minimal aEGFR-EVs content, resulting in the generation of simulated plasma samples with known amounts of aEGFR for our assessment. In Fig. 4b, we assessed the minimum detectable quantity of aEGFR by analyzing simulated plasma samples containing known amounts of aEGFR fraction and comparing them to the estimated amounts obtained from our platform. The universal calibration curve successfully quantified aEGFR+ CD63 EVs at levels as low as 0.01%. Additionally, various fractions aligned well with our theoretical trend, highlighting that aEGFR fractions can be directly quantified from plasma without the requirement of isolation steps such as ultracentrifugation.

### Clinical sample analysis

**Fig. 5:**
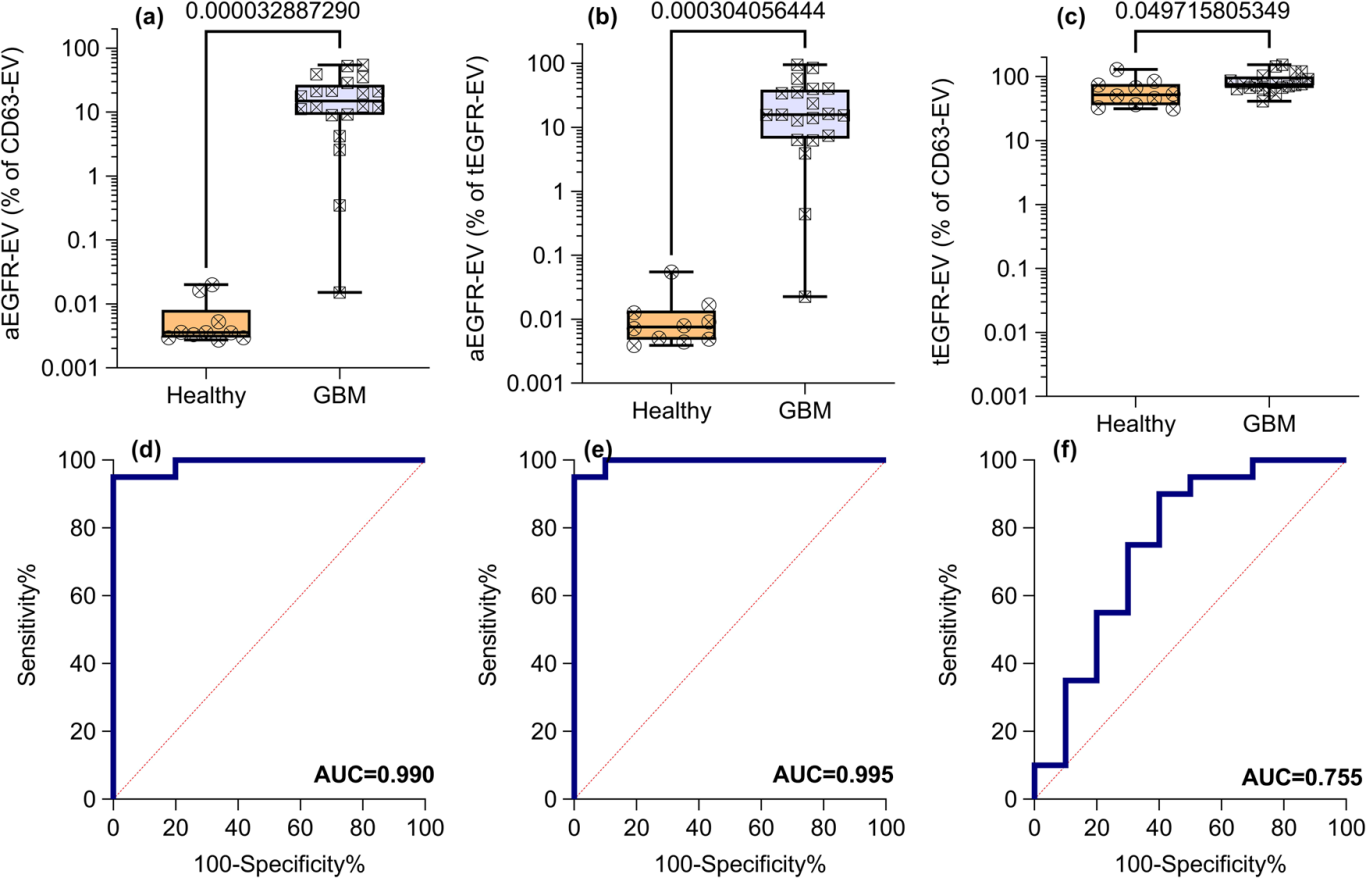
Active and Total EGFR-positive sEV concentrations in healthy humans and subjects with glioblastoma. **a**, **d** The expression and AUC of aEGFR-EVs (normalized to CD63-EVs) with *p*-value = 3.3E-5, (**b**, **e**) aEGFR-EVs (normalized to tEGFR EVs) with *p*-value = 3E-4 and (**c**, **f**) tEGFR-EVs (normalized to CD63-EVs) with *p*-value = 4.97E-2. All reported *p*-values were calculated using a parametric test with Welsh’s correction. (**a**–**c**) are represented using box and whiskers plot where box represents 25^th^ to 75^th^ percentile while whiskers represent the total range of the data. Error bars are one standard deviation. Human subjects include 10 healthy and 20 patients with GBM.

Having established the robustness of the voltage signal for normalized colocalization assay of raw plasma EVs, we test its capability to differentiate patients with glioblastoma multiforme (GBM) and healthy plasma samples. The human plasma samples from the twenty patients with GBM cancer and five healthy subjects were used for analysis. Their fractions of EVs with aEGFR yield p-value of 3.3E-5 (Figs. 5a) and 3E-4 (Fig. 5b) respectively, when normalized with respect to CD63 and tEGFR, with an AUC of 0.99 for both (Fig. 5d,e). The patient samples have an aEGFR-CD63 EV fraction ranging from 0.01% to 60%, a 4-decade variation. The fraction for healthy samples is close to the LOD of 0.01%, suggesting an undetectable amount of aEGFR by the sensor. In contrast, the fraction of tEGFR EVs in GBM plasma (normalized to CD63 EVs) gave a p-value of 4.97E-2 (Fig. 5c) and AUC of 0.755 (Fig. 5f) with a net variation from 25% to slightly over 100% (corresponding to samples that contain EVs with one copy of CD63 and one or more copies of tEGFR). This is a substantial difference from aEGFR measurements that not only spanned several orders of magnitude but was also below the level of detection in healthy patients when normalized relative to CD63 or tEGFR. This result agrees with the previously reported findings that patients with glioblastoma cancer express more variant III EGFR EVs^36^, which is a subset of overall active EGFR.

**Fig. 6:**
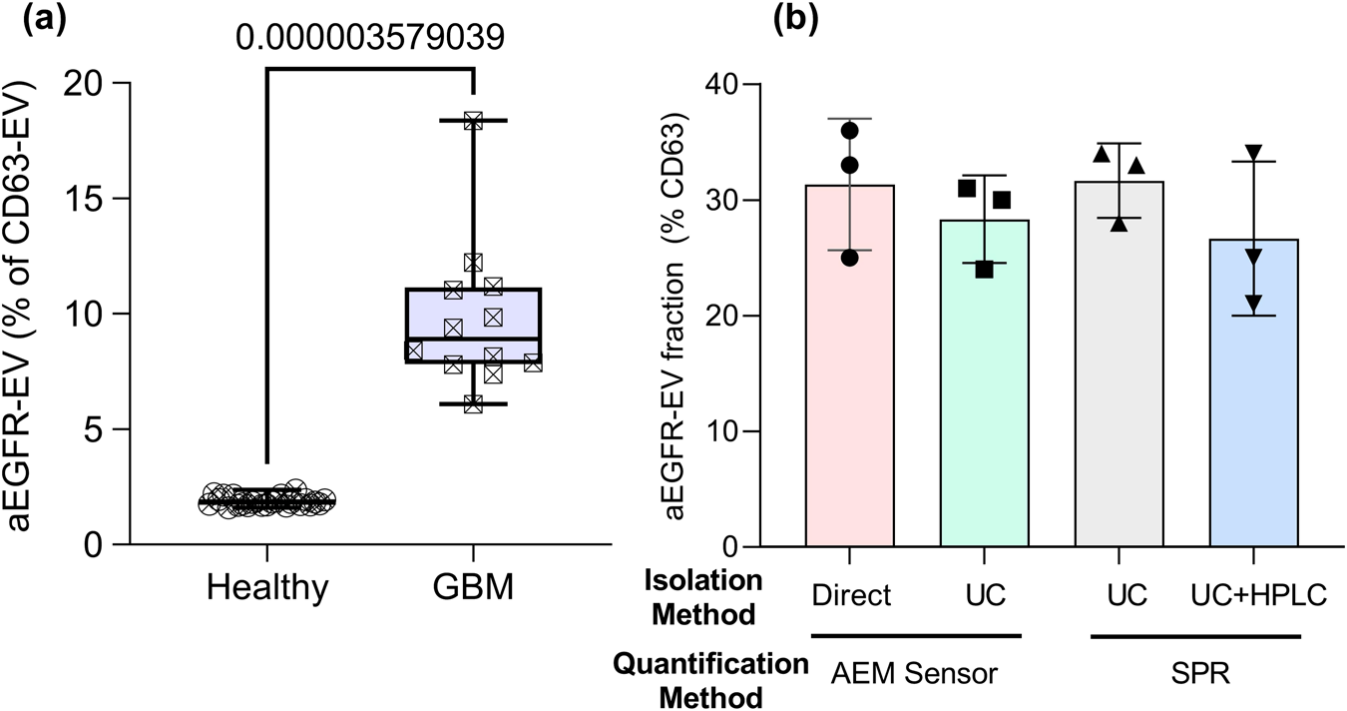
Orthogonal benchmarking of our sensor against Ultracentrifugation and/or HPLC-isolated sEVs using Surface Plasmon Resonance (SPR). **a** Box and whisker plot showing percentage aEGFR in DiFi from different isolation and quantification methods. Subjects include 25 healthy and 12 patients with glioblastoma. Box represents 25^th^ to 75^th^ percentile while whiskers show the range of data. **b** Bar plot showing DiFi-derived sEV aEGFR fraction of ultracentrifugation (UC) isolated DiFi EVs measured in triplicates using Surface Plasmon Resonance (SPR). Error bars are one standard deviation.

We validated the performance of the AEM sensor by comparing the percentage of aEGFR+ CD63 EVs in both untreated and treated DiFi samples. The pretreatment involves two ultracentrifugation (UC) steps at 167000 g for 4–8 h each, a standard protocol to isolate EVs by UC. Figure 6a clearly shows consistent AEM estimate of the aEGFR+ fraction for both treated and untreated DiFi samples. We also benchmarked the AEM results for DiFi against an orthogonal technique, Surface Plasmon Resonance (SPR) with CD63 capture antibodies. Mass-based SPR requires UC pretreatment to remove non-targets. We hence conducted UC for one and UC plus a high-performance liquid chromatograph (HPLC) for the other. The aEGFR fractions measured by SPR for both pretreated samples are consistent with the AEM measurements, although larger error bars are found with the two pretreatment steps. Finally, we benchmarked the AEM sensor data for untreated clinical plasma samples against SPR data after UC pretreatment of the same clinical sample. Consistent results are observed between the SPR data in Fig. 6b (AUC = 1.00) and the AEM data of Fig. 5a (AUC = 0.99).

## Conclusions

In summary, we have successfully developed an automated, low-cost (<$2 in material), and rapid ( < 1 hour) AEM biosensor platform to quantify the fraction of CD63 EVs with a specific cell-surface marker, aEGFR, despite its low abundance and high $${K}_{D}$$. It is the first colocalization EV assay for untreated plasma. The sensor is optimized for EVs, with proper sensor dimension to reduce incubation time without diminishing sensitivity and robustness. The field from a highly charged silica nanoparticle is amplified by long-range ion depletion action of the ion-selective membrane, to affect the electrokinetics at the membrane surface that controls the ion flux, thus producing orders of magnitude amplification of the voltage signal without distortion despite variations in the EV size. Controlled PBS wash with high drag on the EV and the nanoparticle reporter is used against multivalent adsorption of non-targets to enhance specificity to the extent that high-loss isolation is not necessary and untreated plasma can be used. The AEM microsensor is easy to fabricate and was modified with capture antibody using conventional coupling chemistry. Elimination of analyte loss and irreversible association yielded a normalized calibration curve for the fraction of CD63 EV with a colocalized protein. This universal calibration curve for the colocalization fraction has a linear range between 0.01 and 100% colocalization (corresponding to 30 to 300000 EVs/$$\mu L$$). EVs quantification of the active EGFR+ EVs in untreated plasma demonstrated that the biosensor is excellent for the detection of specific types of cancer. Glioblastoma clinical sample analysis showed a clear differentiation (AUC ~ 0.99) between patients with cancer and healthy subjects with a single marker colocalization assay, with a *p*-value of 0.000033 that is superior to any other EV diagnostics.

The current platform could be used to follow possible residual or recurrent GBM and other cancers with amplified aEGFR and tEGFR, as the presence of amplification/mutation of EGFR is frequently retained in metastatic disease. Its use in a screening or diagnostic test to identify cancer type, location, and stage is more complex. Other cancers, like colorectal cancer, can have EGFR amplification and can show an enhanced EGFR signal in plasma from these patients^6^. Therefore, such an EGFR active and total signature might not necessarily indicate the presence of GBM specifically. Likewise, patients with GBM can have amplified or mutated EGFR but can also have non-EGFR driven forms of the disease. To establish this as a specific test for a specific cancer would require analyzing a much larger set of GBM cases matched to EGFR status along with testing plasma from other cancers and diseases that might regulate biofluid EGFR. Each distinct tumour type or genotype might regulate the amount of aEGFR, tEGFR, and CD63 EV-carrying forms of such EGFR analytes. Therefore, based on these measures alone it might be possible to tell the difference between different kinds of GBMs and different kinds of diseases that regulate these EV-carried proteins. More broadly, a multi-biomarker version of this platform, which includes markers other than aEGFR or tEGFR and reference EV markers beyond CD63, should enhance the specificity of the screening test. The current diagnostic platform can be scaled up for such large-library testing of untreated plasma from a large cohort of cancer patients to establish specific profiles for different cancers at different stages.

## Methods

### Ethics statement and human plasma samples

We received Glioblastoma and control group samples from Precision for Medicine and Tumour Targeting Laboratory, ONJCRI, Melbourne, Australia. An approved IRB protocol is already in place at Precision for the collection of plasma samples from patients. Precision for Medicine works with regulatory authorities and accrediting organizations around the world to ensure that the sample collection process and protocol follow the latest FDA, EMA, and MHRA guidelines. The pooled healthy plasma samples were obtained from Innovative Research (IPLAK2E10ML). Out of 20 GBM plasma clinical samples that we tested, four GBM were purchased from Precision for Medicine and the remaining 16 samples were received from Andrew Scott and Hui Gan, Tumour Targeting Laboratory, ONJCRI, Melbourne, Australia. 10 healthy plasma samples were purchased from Precision for Medicine. The gender, age at diagnosis, sample collection time point and key tumour pathology results of the tested GBM samples are listed in the Supplementary Table 1.

The tested samples of blood were collected from consented adult patients diagnosed with brain cancer at different timepoints of their disease. The blood was then processed within 2 h of collection for platelet-free plasma and then stored at −70 °C or lower until analysis.

The standard pathology testing of patient tumour samples were performed that includes IDH1 wild type/ Mutation, ATRX (present/mutant), Ki67, p53 positivity, GFAP, Olig2 and MGMT methylation status. Through involvement in particular trials or NGS panel assessments, EGFRvIII presence and EGFR amplification, respectively, were determined in some samples. The studies involving human participants were reviewed and approved by the Institutional Ethics Committee (IEC) at Austin Health, Melbourne, Australia. The patients/participants provided their written informed consent to participate in this study. All ethical regulations relevant to human research participants were followed.

### Fabrication of anion-exchange membrane (AEM) sensor

The anion exchange membrane (AEM) composed of polystyrene-divinylbenzene fine particles with strong basic quaternary ammonium groups (R-(CH_3_)_3_N^+^) supported by polyethylene as a binder and polyamide/polyester textile fiber was obtained from the Mega a.s. (Straz pod Ralskem, Czech Republic) and used as a sensor. The AEM sensor was fabricated by embedding a small piece of anion exchange membrane of approximate dimensions 0.3 mm × 0.9 mm in an epoxy resin (TAP Quik-Cast, Tap plastic) using an optimized reported protocol^31^. Briefly, the membrane was hand-cut and placed on the tip of a silicone mold. A glass slide was then placed on top of the membrane piece. A two-component (side A and side B, 1:1 ratio) epoxy polyurethane resin mixture was then pushed to embed the AEM sensor^31^. A 3D-printed sensor reservoir was then attached to the sensor disc using the same polyurethane resin mixture. The prepared sensor was soaked in de-ionized (DI) water overnight before further use.

### Fabrication of biochip

A 25 mm × 54 mm (w × l) size biochip having a microfluidic channel of 3 mm × 35 mm × 250 µm (w × l × h) was fabricated as shown in Fig. 1. Three layers of polycarbonate sheet of 0.3 mm thickness were used to fabricate using our reported protocol^31^. Briefly, the sheets with orifices for the inlet, outlet, sensor, and channel were cut using a cutting plotter (FC700. Graphtec Corp., Japan). The sheets were then thermally bonded in an oven (Fisher Scientific, Isotemp Oven) at 177 ˚C for 15 min. The small pieces of tubes for the inlet and outlet and three different-sized tubes were attached in between the inlet and outlet for mounting a sensor and different electrodes using Acrifix UV glue. The electrode reservoirs were filled with 2% agarose gel to create a barrier between the microfluidic channel and the reservoirs.

### Functionalization of the antibody on the AEM surface

The surface modification of the anion-exchange membrane and antibody functionalization followed a previously optimized protocol^31^. Initially, the membrane surface was treated with a 0.1 M solution of 3,3’,4,4’-Benzophenonetetracarboxylic acid (Sigma-Aldrich, USA) at pH 7 for 10 min. Subsequently, the surface was exposed to UV light at 365 nm (using an Intelli ray 600 shuttered UV floodlight) for 90 s while purging with N2 gas. The sensor was then rinsed with deionized (DI) water. This surface modification process was repeated three times to ensure the generation of an adequate number of –COOH groups on the membrane surface. Following that, the sensors were immersed in DI water at pH 2 for 5 h and subsequently washed with 0.1X PBS at pH 7. The carboxylated membrane surface was then ready for functionalization with the antibody probe using EDC (Thermo Fisher, USA) coupling chemistry. A 20 µL solution of 0.4 M EDC in 50 mM MES buffer at pH 6 was applied to the sensor surface for 40 min. The solution was then removed, and the sensor was washed with 1X PBS. Finally, a solution of the probe antibody (0.1 mg/mL) was incubated overnight on the sensor surface at four-degree celcius^31^.

### Conjugation of antibody to silica reporter particles

To prepare the carboxylated silica particles, 500 µl of 2.5% silica particles with a size of 50 nm (Microspheres-Nanospheres (New York, USA)) were mixed with 500 µl of 1X PBS. The mixture was then centrifuged at 17,000 g for 10 min, and the supernatant was discarded. This washing process was repeated twice. After the final wash, the particles were resuspended in 1 ml of 50 mM MES buffer at pH 6. Next, a solution containing equal volumes of 200 mM EDC and 200 mM sulfo-NHS (EMD Millipore, USA) prepared in MES buffer was added to the silica particles. The mixture was stirred for 1 hour at room temperature. Subsequently, the silica particles were washed three times with 1X PBS by centrifugation at 17,000 g for 10 min each time. Finally, 20 µL of a 0.1 mg/mL solution of the detection antibody was added to the 1 mL of silica particles in 1X PBS. The mixture was mixed overnight at 150 RPM at 4 °C. Afterward, the particles were washed three times with 1X PBS to remove any unbound antibodies from the solution.

### Labelling of anti-CD63 antibody

To confirm the binding of antibodies on the sensor and silica reporters, the anti-CD63 antibody was labelled using Zip Alexa Fluor 488 rapid antibody labelling kit (ThermoFisher, USA) according to the manufacturer’s instructions with some modifications. In brief, 10 µl of 1 M sodium bicarbonate solution was added to the 100 µl of 0.5 mg/mL antibody solution. The antibody solution was then added to the Alexa Fluor dye. The solution was mixed using a micropipette and incubated for 15 min at room temperature. The free dye was removed using Amicon Ultra 100k centrifugal filter devices and the labelled antibody was stored at 4 °C till further used.

### Voltage signal measurements

The current-voltage characteristics (CVC) signal of the AEM sensor was evaluated using a Gamry Potentiostat/Galvanostat/ZRA (Reference 600, Gamry Instruments Inc., USA) in a four-electrode configuration. Crocodile clips were used to connect the electrodes to the instrument, and they were securely mounted on the biochip. The current was supplied through the two platinum electrodes, while the potential across the membrane was measured using Ag/AgCl reference electrodes (World Precision Instruments, USA). CVC measurements were conducted in a 0.1X PBS solution. The current was incrementally increased from 0 µA to double the limiting current, and the potential was recorded at a step rate of 1 µA/s. The measurements were performed using Gamry Framework software, and the obtained spectra were analysed using Gamry Echem Analyst software. To introduce different buffers into the biochip, a custom-made microfluidic pump was utilized. The pump, controlled by a computer, allowed for the selection of various buffers and adjustable flow rates. A visual representation of the pump is presented in Fig. 1.

### Isolation of extracellular vesicles from cell culture media

To optimize different sensing parameters for EV detection, EVs were isolated from the human colorectal cancer cell line, DiFi using ultracentrifugation. Human DiFi cells were cultured as previously described^38^. The cell culture media was centrifuged at 250 g and then 2500 g both for 10 min in order to remove cellular debris and the supernatant was collected in a new vial. Then the supernatant was filtered through a 0.22 μm polyethersulfone filter (Nalgene) to remove the microparticles by gravity flow. Further, the collected filtrate was concentrated by a 100,000 molecular-weight cutoff (Millipore) centrifugal concentrator. Finally, the high-speed centrifugation at 167,000 g for 4 h in an SW32 Ti swinging-bucket rotor (Beckman Coulter) was used to separate and further wash the EV from the concentrate collected in the previous step. The EVs suspended in PBS 25 mM HEPES were used immediately or stored at −80 °C in aliquots for further use. The average particle size and particle concentration were measured using the nanoparticle tracking analysis (NTA) method (NanoSight^TM^ NS300, Malvern Instruments Ltd., UK). Similar approach was taken for HCA7-derived CC-CR cell line (colorectal cancer cell line). Study was performed according to the Minimal Information for Studies of Extracellular Vesicles 2018 guidelines^39^.

### Antibodies

Purified mouse anti-human CD63 antibody (Catalog No. 556019) and isotype control antibody (Catalog No. 555746) were bought from BD Biosciences, USA. Mab806 antibody (ABT-806, Catalog No. TAB-228CL) and Human EGFR (cetuximab) antibody (Catalog No. MAB9577) were purchased from Creative Biolabs, USA, and R&D Systems respectively.

### ELISA

Maxisorp 96-well plate (Nunc) was coated with an anti-CD63 antibody by incubating overnight at 4 °C. Then, the plate was washed with 1X PBS. The plate was blocked using 2% BSA in a blocking solution for 1 h at room temperature. EV samples of 100 µL were added to each well and incubated for 2.5 h at room temperature. After washing, 100 µL of biotinylated anti-CD63 antibody was added to each well for 1 h at room temperature. Then, 100 µL of HRP-Streptavidin molecules were added to each well and incubated for 45 min at room temperature. The absorbance signal was measured at 450 nm using a plate reader.

### Statistics and reproducibility

The all experiments are performed in triplicate. The standard errors (described in figure captions) of all datasets are calculated and plotted using the software GraphPad Prism and OriginPro and double-checked manually. Data are shown as individual data points and mean ± error with sample size described in figure captions.

### Reporting summary

Further information on research design is available in the Nature Portfolio Reporting Summary linked to this article.

## Supplementary information

Supplementary Information

Description of Additional Supplementary File

Supplementary Data 1

Reporting Summary

## Data Availability

Source data for graphs and charts can be found in Supplementary Data 1 associated with this article. Additional data that contributed to this study are present in the Supplementary Information.

## References

[1] Kalluri, R. & LeBleu, V. S. The biology, function, and biomedical applications of exosomes. *Science* **367**, eaau6977 (2020). 32029601 10.1126/science.aau6977 PMC7717626

[2] Boriachek, K. et al. Avoiding Pre-Isolation Step in Exosome Analysis: Direct Isolation and Sensitive Detection of Exosomes Using Gold-Loaded Nanoporous Ferric Oxide Nanozymes. *Anal. Chem.* **91**, 3827–3834 (2019). 30735354 10.1021/acs.analchem.8b03619

[3] Morhayim, J., Rudjito, R., van Leeuwen, J. P. & van Driel, M. Paracrine Signaling by Extracellular Vesicles via Osteoblasts. *Curr. Mol. Biol. Rep.* **2**, 48–55 (2016). 27429899 10.1007/s40610-016-0034-6 PMC4922391

[4] Cheng, N. et al. Recent Advances in Biosensors for Detecting Cancer-Derived Exosomes. *Trends Biotechnol.* **37**, 1236–1254 (2019). 31104858 10.1016/j.tibtech.2019.04.008

[5] Chao, G., Cochran, J. R. & Wittrup, K. D. Fine epitope mapping of anti-epidermal growth factor receptor antibodies through random mutagenesis and yeast surface display. *J. Mol. Biol.* **342**, 539–550 (2004). 15327953 10.1016/j.jmb.2004.07.053

[6] Higginbotham, J. N. et al. Identification and characterization of EGF receptor in individual exosomes by fluorescence-activated vesicle sorting. *J. Extracell. Vesicles* **5**, 29254 (2016). 27345057 10.3402/jev.v5.29254 PMC4921784

[7] Johns, T. G. et al. Identification of the epitope for the epidermal growth factor receptor-specific monoclonal antibody 806 reveals that it preferentially recognizes an untethered form of the receptor. *J. Biol. Chem.* **279**, 30375–30384 (2004). 15075331 10.1074/jbc.M401218200

[8] Sivasubramanian, A., Chao, G., Pressler, H. M., Wittrup, K. D. & Gray, J. J. Structural model of the mAb 806-EGFR complex using computational docking followed by computational and experimental mutagenesis. *Structure* **14**, 401–414 (2006). 16531225 10.1016/j.str.2005.11.022

[9] Garrett, T. P. et al. Antibodies specifically targeting a locally misfolded region of tumor associated EGFR. *Proc. Natl Acad. Sci. USA* **106**, 5082–5087 (2009). 19289842 10.1073/pnas.0811559106 PMC2656555

[10] Gan, H., Burgess, A. W., Clayton, A. H. A. & Scott, A. M. Targeting a conformationally exposed tumor specific epitope of EGFR as a strategy for cancer therapy. *Cancer Res.* **72**, 2924–2930 (2012). 20212. 22659454 10.1158/0008-5472.CAN-11-3898

[11] Alsaif, M. et al. Analysis of serum and plasma identifies differences in molecular coverage, measurement variability, and candidate biomarker selection. *Proteom. Clin. Appl.* **6**, 297–303 (2012). 10.1002/prca.201100061 22641612

[12] Orosz, F. & Ovadi, J. A simple method for the determination of dissociation constants by displacement ELISA. *J. Immunol. Methods* **270**, 155–162 (2002). 12379321 10.1016/s0022-1759(02)00295-8

[13] Heinrich, L., Tissot, N., Hartmann, D. J. & Cohen, R. Comparison of the results obtained by ELISA and surface plasmon resonance for the determination of antibody affinity. *J. Immunol. Methods* **352**, 13–22 (2010). 19854197 10.1016/j.jim.2009.10.002

[14] Wang, C., Senapati, S. & Chang, H. C. Liquid biopsy technologies based on membrane microfluidics: High-yield purification and selective quantification of biomarkers in nanocarriers. *Electrophoresis* **41**, 1878–1892 (2020). 32180242 10.1002/elps.202000015 PMC7492446

[15] Qinsi, Z. et al. Ultra-stable organic fluorophores for single-molecule research. *Chem. Soc. Rev.* **43**, 1044–1056 (2014). 24177677 10.1039/c3cs60237k PMC3946787

[16] Roth, S. et al. Improving the Sensitivity of Fluorescence-Based Immunoassays by Photobleaching the Autofluorescence of Magnetic Beads. *Small* **15**, e1803751 (2019). 30411493 10.1002/smll.201803751

[17] Ou, X. Y. et al. Autofluorescence-Free Immunoassay Using X-ray Scintillating Nanotags. *Anal. Chem.* **90**, 6992–6997 (2018). 29757612 10.1021/acs.analchem.8b01315

[18] Mizenko, R. R. et al. Tetraspanins are unevenly distributed across single extracellular vesicles and bias sensitivity to multiplexed cancer biomarkers. *J. Nanobiotechnol.* **19**, 250 (2021). 10.1186/s12951-021-00987-1 PMC8379740 34419056

[19] Moura, S. L., Martín, C. G., Martí, M. & Pividori, M. I. Electrochemical immunosensing of nanovesicles as biomarkers for breast cancer. *Biosens. Bioelectron.* **150**, 111882 (2020). 31786017 10.1016/j.bios.2019.111882

[20] Wei, J. et al. Triple-color fluorescence co-localization of PD-L1-overexpressing cancer exosomes. *Microchimica Acta* **189**, 182 (2022). 35394232 10.1007/s00604-022-05278-6

[21] Miao, Y. & Liao, J. K. Potential serum biomarkers in the pathophysiological processes of stroke. *Expert Rev. Neurother.* **14**, 173–185 (2014). 24417214 10.1586/14737175.2014.875471 PMC3939679

[22] Cekic, S., Zlatanovic, G., Cvetkovic, T. & Petrovic, B. Oxidative stress in cataractogenesis. *Bosn. J. Basic Med Sci.* **10**, 265–269 (2010). 20846136 10.17305/bjbms.2010.2698 PMC5504506

[23] Koppel, J. L., Mueller, D. & Olwin, J. H. Nature of the inhibition of thrombin formation by sulfhydryl-oxidizing agents. *Am. J. Physiol.* **187**, 113–121 (1956). 13362599 10.1152/ajplegacy.1956.187.1.113

[24] Slouka, Z., Senapati, S., Yan, Y. & Chang, H. C. Charge inversion, water splitting, and vortex suppression due to DNA sorption on ion-selective membranes and their ion-current signatures. *Langmuir* **29**, 8275–8283 (2013). 23742037 10.1021/la4007179

[25] Chang, H.-C., Yossifon, G. & Demekhin, E. A. Nanoscale Electrokinetics and Microvortices: How Microhydrodynamics Affects Nanofluidic Ion Flux. *Annu. Rev. Fluid Mech.* **44**, 401–426 (2012).

[26] Sensale, S., Ramshani, Z., Senapati, S. & Chang, H. C. Universal Features of Non-equilibrium Ionic Currents through Perm-Selective Membranes: Gating by Charged Nanoparticles/Macromolecules for Robust Biosensing Applications. *J. Phys. Chem. B* **125**, 1906–1915 (2021). 33410691 10.1021/acs.jpcb.0c09916

[27] Slouka, Z., Senapati, S. & Chang, H. C. Microfluidic systems with ion-selective membranes. *Annu Rev. Anal. Chem. (Palo Alto Calif.)* **7**, 317–335 (2014). 24818814 10.1146/annurev-anchem-071213-020155

[28] Kumar, S., Maniya, N., Wang, C., Senapati, S. & Chang, H.-C. Quantifying PON1 on HDL with nanoparticle-gated electrokinetic membrane sensor for accurate cardiovascular risk assessment. *Nat. Commun.* **14**, 557 (2023). 36732521 10.1038/s41467-023-36258-w PMC9895453

[29] Mathieu, M. et al. Specificities of exosome versus small ectosome secretion revealed by live intracellular tracking of CD63 and CD9. *Nat. Commun.* **12**, 4389 (2021). 34282141 10.1038/s41467-021-24384-2 PMC8289845

[30] Li, D., Wang, C., Sun, G., Senapati, S. & Chang, H. C. A shear-enhanced CNT-assembly nanosensor platform for ultra-sensitive and selective protein detection. *Biosens. Bioelectron.* **97**, 143–149 (2017). 28587929 10.1016/j.bios.2017.05.053

[31] Ramshani, Z. et al. A multiplexed immuno-sensor for on-line and automated monitoring of tissue culture protein biomarkers. *Talanta* **225**, 122021 (2021). 33592751 10.1016/j.talanta.2020.122021

[32] Senapati, S. et al. An ion-exchange nanomembrane sensor for detection of nucleic acids using a surface charge inversion phenomenon. *Biosens. Bioelectron.* **60**, 92–100 (2014). 24787123 10.1016/j.bios.2014.04.008 PMC4445831

[33] Ramshani, Z. et al. Extracellular vesicle microRNA quantification from plasma using an integrated microfluidic device. *Commun. Biol.* **2**, 189 (2019). 31123713 10.1038/s42003-019-0435-1 PMC6527557

[34] Kim, K. M. et al. Surface treatment of silica nanoparticles for stable and charge-controlled colloidal silica. *Int J. Nanomed.* **9**, 29–40 (2014). 10.2147/IJN.S57922 PMC4279762 25565824

[35] Im, H. et al. Label-free detection and molecular profiling of exosomes with a nano-plasmonic sensor. *Nat. Biotechnol.* **32**, 490–495 (2014). 24752081 10.1038/nbt.2886 PMC4356947

[36] Kilic, T. et al. Multielectrode Spectroscopy Enables Rapid and Sensitive Molecular Profiling of Extracellular Vesicles. *ACS Cent. Sci.* **8**, 110–117 (2022). 35111901 10.1021/acscentsci.1c01193 PMC8802188

[37] Chang, H. –C.; Yeo. L., *Electrokinetically driven microfluidics and nanofluidics*. Cambridge university press, 2010.

[38] Zhang, Q. et al. Supermeres are functional extracellular nanoparticles replete with disease biomarkers and therapeutic targets. *Nat. Cell Biol.* **23**, 1240–1254 (2021). 34887515 10.1038/s41556-021-00805-8 PMC8656144

[39] Théry, C. et al. Minimal information for studies of extracellular vesicles 2018 (MISEV2018): a position statement of the International Society for Extracellular Vesicles and update of the MISEV2014 guidelines. *J. Extracell. Vesicles* **7**, 1535750 (2018). 30637094 10.1080/20013078.2018.1535750 PMC6322352

