# Supplementary material for: An anion exchange membrane sensor detects EGFR and its activity state in plasma CD63 extracellular vesicles from patients with glioblastoma
Source: Commun Biol. 2024 Jun 3;7:677. doi: 10.1038/s42003-024-06385-1 (PMC11148014; doi:10.1038/s42003-024-06385-1)
Supplement: Supplementary file 1 — Supplementary Information [file 42003_2024_6385_MOESM1_ESM.pdf]

Supplementary Information

An Anion Exchange Membrane Sensor detects EGFR and its Activity State in Plasma CD63 Extracellular Vesicles from Patients with Glioblastoma

Nalin H. Maniya[1†], Sonu Kumar[1†], Jeffrey L. Franklin[2,3], James N. Higginbotham[2], Andrew M Scott[4,5,6,7], Hui K Gan[4,6,7], Robert J. Coffey[2,3], Satyajyoti Senapati[1]*, Hsueh-Chia Chang[1]*

[1]Department of Chemical and Biomolecular Engineering, University of Notre Dame, Notre Dame, IN 46556, USA,

[2]Department of Medicine, Vanderbilt University Medical Center, Nashville, TN 37232, USA.

[3]Department of Cell and Developmental Biology, Vanderbilt University School of Medicine, Nashville, TN 37232, USA

[4]Tumour Targeting Laboratory, Olivia Newton-John Cancer Research Institute, Melbourne, Victoria, Australia.

[5]Department of Molecular Imaging and Therapy, Austin Health, Melbourne, Victoria, Australia.

[6]School of Cancer Medicine, La Trobe University, Melbourne, Victoria, Australia.

[7]Department of Medicine, University of Melbourne, Melbourne, Victoria, Australia.

[†] Authors contributed equally to the work.

*Corresponding author: hchang@nd.edu (H.-C. Chang), ssenapat@nd.edu (S. Senapati)

The supplemental information provides additional details on the following.

Supplementary Figure 1. Photograph of (a) cut anion-exchange membrane (AEM), (b) top and bottom parts, and (c) fully prepared AEM sensor.

Supplementary Figure 2. (a) AEM sensor functionalization using Alexa fluor 488 labeled CD63 antibody and (b) Silica reporter particles functionalization using Alexa fluor 488 labeled CD63.

Supplementary Figure 3. NTA spectrum of isolated EVs. The average hydrodynamic diameter and concentration of the EVs were found to be 117 nm and 1×10^{10} particles/mL, respectively.

Supplementary Figure 4. CD63 ELISA of different concentrations of isolated DiFi EVs.

Supplementary Table 1. Showing the gender, age at diagnosis, sample collection point and disease stage of the tested GBM samples

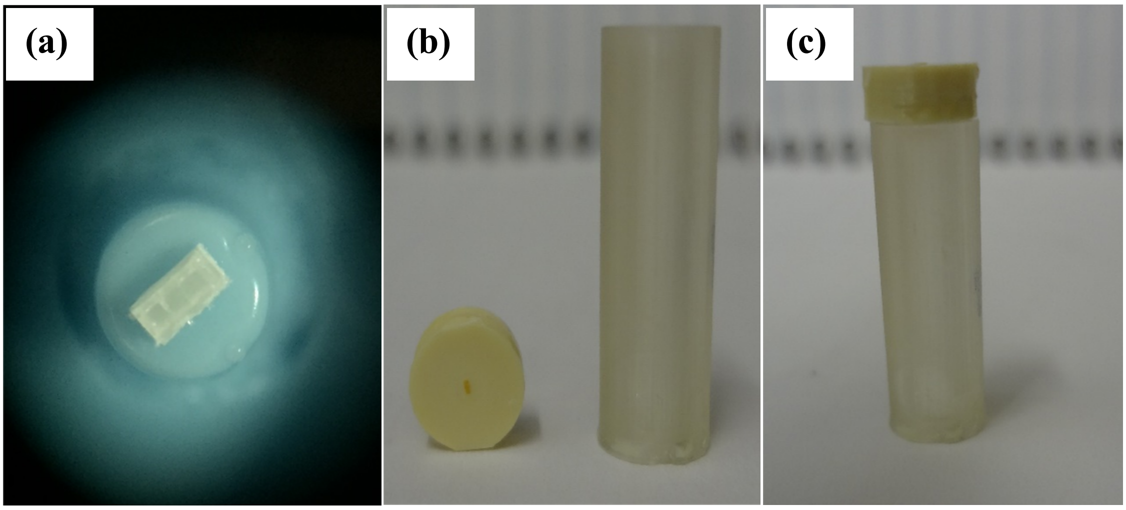

Supplementary Figure 1. Photograph of (a) cut anion-exchange membrane (AEM), (b) top and bottom parts, and (c) fully prepared AEM sensor.

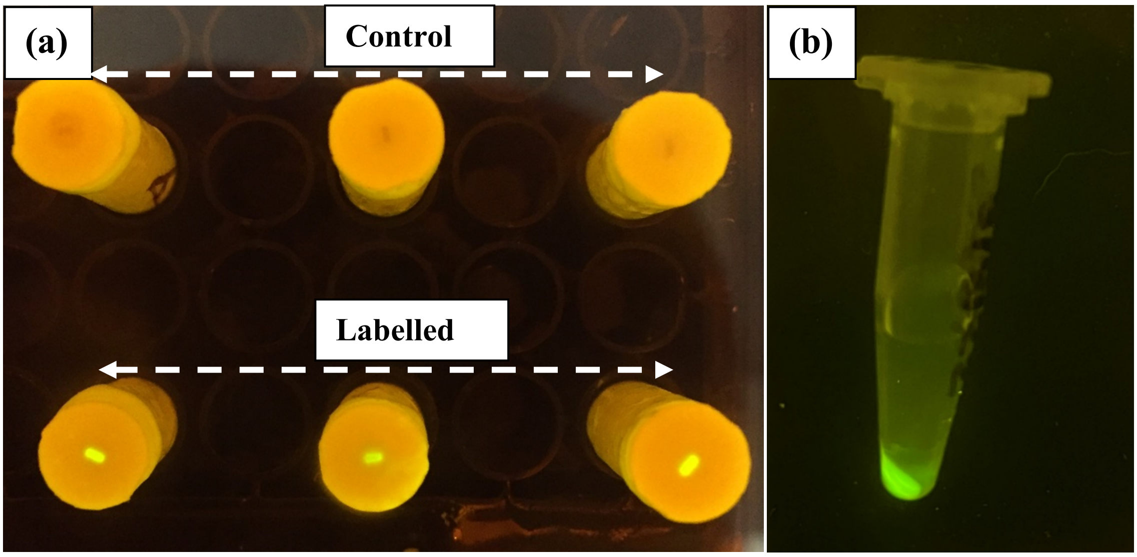

Supplementary Figure 2. (a) AEM sensor functionalization using Alexa fluor 488 labeled CD63 antibody and (b) Silica reporter particles functionalization using Alexa fluor 488 labeled CD63.

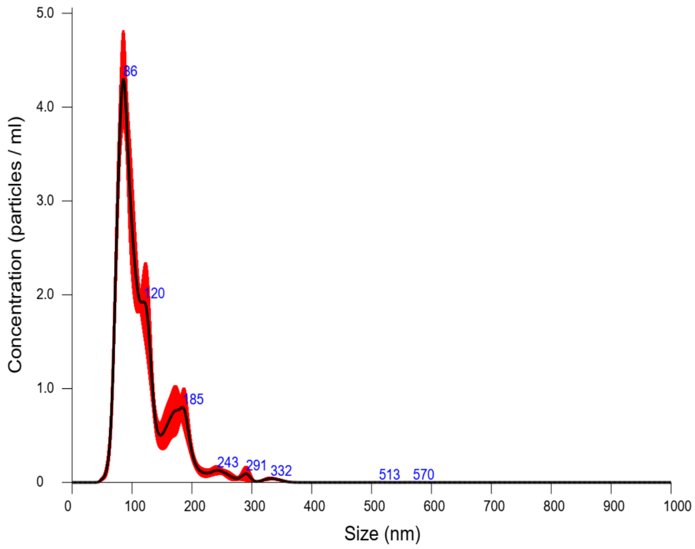

Supplementary Figure 3. NTA spectrum of isolated EVs. The average hydrodynamic diameter and concentration of the EVs were found to be 117 nm and 1×10^{10} particles/mL, respectively.

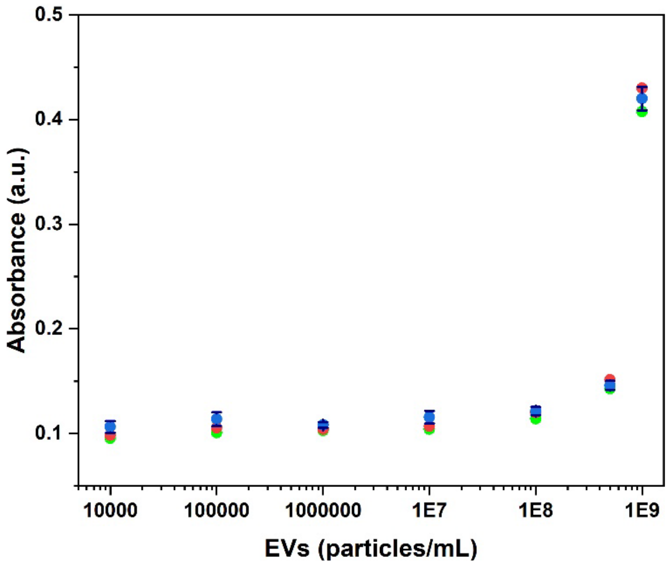

Supplementary Figure 4. CD63 ELISA of different concentrations of isolated DiFi EVs. Error bars indicate the standard deviation (SD) in plot. Derived from one biological replicate.

Supplementary Table 1. Showing the gender, age at diagnosis, sample collection point and disease stage of the tested GBM samples

Sample received from Andrew Scott and Hui Gan, Tumour Targeting Laboratory, ONJCRI, Melbourne, Australia				
Sample No.	Gender	Age at diagnosis	Sample collection time point*	Key tumour pathology results
1.	Male	51	After first-line chemo-radiation in STUPP but before adjuvant chemotherapy	GBM: IDH WT, ATRX pres, p53 5%, (+meningioma) EGFR unknown, MGMT methylated, Ki67 5%, GFAP+
2.	Male	77	At the time of tumour progression after first line chemo-radiation/adjuvant (STUPP tmt)	GBM: IDH WT, ATRX pres, EGFR unknown, MGMT unmethylated, p53>80%, Ki67 40%, GFAP+
3.	Male	54	At the time of tumour progression after first line chemo-radiation/adjuvant (STUPP tmt)	GBM: IDH WT, ATRX pres, p53 20%, MGMT meth, EGFRvIII-ve, EGFR amp
4.	Female	38	At the start of Chemo-radiation treatment (following surgery)	GBM: IDH WT, EGFR non-amp, no BRAF mut, no TRT prom mut, no H3F3A mut
5.	Female	77	At the time of tumour progression after first line chemo-radiation/adjuvant (STUPP tmt)	GBM: IDH1 WT, ATRX pres, MGMT meth, p53 15-20%, GFAP+
6.	Female	38	Following 2nd disease progression, during 3rd line treatment	GBM: IDH1 WT, ATRX pres, MGMT meth, p53 20%, EGFR amp
7.	Female	64	Before the start of Chemo-radiation treatment (following surgery)	GBM: IDH1 MUT, ATRX pres, MGMT meth, p53 +, EGFR amp, GFAP+, Oligo2 +, Ki67 25%
8.	Female	71	Following 1st disease progression, and before 2nd line treatment	GBM: IDH1 WT, ATRX pres, MGMT unmeth, p53 90%, Ki67 60%, GFAP+
9.	Male	39	Before the start of Chemo-radiation treatment (following surgery)	GBM: IDH1 WT, ATRX pres, MGMT meth, p53 +, Ki67 10%, weak Oligo 2, GFAP+
10.	Male	56	Before the start of Chemo-radiation treatment (following surgery)	GBM: IDH1 WT, ATRX pres, MGMT unknown, p53 +, GFAP+, Oligo2 +, Ki67 15-20%
11.	Female	44	Prior to surgery for resection of primary tumour	GBM: IDH1 MUT, MGMT meth, p53 +, GFAP+, Oligo2 +, Ki67 40%
12.	Female	33	Before the start of Chemo-radiation treatment (following surgery)	GBM: ATRX MUT, MGMT unmeth, p53-ve, GFAP+, Oligo2 +, Ki67 5%
13.	Female	46	Prior to surgery for resection of primary tumour	GBM: IDH1 WT, ATRX pres, MGMT unmeth, p53+, EGFR amp, GFAP+, Oligo2 +, Ki67 30%
14.	Female	46	Following 2nd disease progression, and before 3rd line treatment	GBM: IDH1 WT, ATRX pres, MGMT unmeth, p53+, EGFR amp, GFAP+, Ki67 40%
15.	Male	54	Prior to surgery for resection of primary tumour	GBM: IDH1 WT, MGMT meth
16.	Male	39	Prior to surgery for resection of primary tumour	GBM: IDH1 WT, ATRX pres, MGMT meth, p53+
Samples acquired from Precision for Medicine				
17.	Female	83	N/A	GBM

18.	Female	81	N/A	GBM: Isocitrate dehydrogenase 1, Negative, O6-methylguanine-DNA methyltransferase promoter methylation, Negative
19.	Male	53	N/A	GBM: 06-methylguanine-DNA methyltransferase (MGMT), Negative, IDH1/2 Mutation, Not Detected, ATRX Immunoreactivity, Positive (Intact)
20.	Female	83	N/A	GBM

*STUPP - standard protocol for brain cancer treatment, begins with CRT, CRT - chemotherapy and radiation treatment combined, PD - Disease Progression, tmt-treatment
