# Supplementary material for: An anion exchange membrane sensor detects EGFR and its activity state in plasma CD63 extracellular vesicles from patients with glioblastoma
Source: Commun Biol. 2024 Jun 3;7:677. doi: 10.1038/s42003-024-06385-1 (PMC11148014; doi:10.1038/s42003-024-06385-1)
Supplement: Supplementary file 2 — Description of Additional Supplementary File [file 42003_2024_6385_MOESM2_ESM.pdf]

Description of Additional Supplementary Files

File name- Supplementary Data 1

File description- The source data behind the graphs of the paper
