# Supplementary material for: An anion exchange membrane sensor detects EGFR and its activity state in plasma CD63 extracellular vesicles from patients with glioblastoma
Source: Commun Biol. 2024 Jun 3;7:677. doi: 10.1038/s42003-024-06385-1 (PMC11148014; doi:10.1038/s42003-024-06385-1)
Supplement: Supplementary file 4 — Reporting Summary [file 42003_2024_6385_MOESM4_ESM.pdf]

nature portfolio

Corresponding author(s): Prof. Hsueh-Chia Chang
Prof. Satyajyoti Senapati

Last updated by author(s): Mar 28, 2024

Reporting Summary

Nature Portfolio wishes to improve the reproducibility of the work that we publish. This form provides structure for consistency and transparency in reporting. For further information on Nature Portfolio policies, see our Editorial Policies and the Editorial Policy Checklist.

Statistics

For all statistical analyses, confirm that the following items are present in the figure legend, table legend, main text, or Methods section.

n/a	Confirmed	
☐	☒	The exact sample size (*n*) for each experimental group/condition, given as a discrete number and unit of measurement
☐	☒	A statement on whether measurements were taken from distinct samples or whether the same sample was measured repeatedly
☐	☒	The statistical test(s) used AND whether they are one- or two-sided<br>*Only common tests should be described solely by name; describe more complex techniques in the Methods section.*
☒	☐	A description of all covariates tested
☒	☐	A description of any assumptions or corrections, such as tests of normality and adjustment for multiple comparisons
☐	☒	A full description of the statistical parameters including central tendency (e.g. means) or other basic estimates (e.g. regression coefficient) AND variation (e.g. standard deviation) or associated estimates of uncertainty (e.g. confidence intervals)
☐	☒	For null hypothesis testing, the test statistic (e.g. *F*, *t*, *r*) with confidence intervals, effect sizes, degrees of freedom and *P* value noted<br>*Give P values as exact values whenever suitable.*
☒	☐	For Bayesian analysis, information on the choice of priors and Markov chain Monte Carlo settings
☒	☐	For hierarchical and complex designs, identification of the appropriate level for tests and full reporting of outcomes
☒	☐	Estimates of effect sizes (e.g. Cohen's *d*, Pearson's *r*), indicating how they were calculated

Our web collection on statistics for biologists contains articles on many of the points above.

Software and code

Policy information about availability of computer code

Data collection	GAMRY Potentiostat Software, NanoSight NTA Software
Data analysis	MATLAB R2020B, GraphPad Prism 9, OriginPro 2020 SR1

For manuscripts utilizing custom algorithms or software that are central to the research but not yet described in published literature, software must be made available to editors and reviewers. We strongly encourage code deposition in a community repository (e.g. GitHub). See the Nature Portfolio guidelines for submitting code & software for further information.

Data

Policy information about availability of data

All manuscripts must include a data availability statement. This statement should provide the following information, where applicable:

- Accession codes, unique identifiers, or web links for publicly available datasets
- A description of any restrictions on data availability
- For clinical datasets or third party data, please ensure that the statement adheres to our policy

All data supporting the findings of this study are available within the paper and its Supplementary Information.

Research involving human participants, their data, or biological material

Policy information about studies with human participants or human data. See also policy information about sex, gender (identity/presentation), and sexual orientation and race, ethnicity and racism.

Reporting on sex and gender	The sample size is not large enough to do gender or sex based analysis.
Reporting on race, ethnicity, or other socially relevant groupings	No socially constructed or socially relevant categorization variable used in the manuscript.
Population characteristics	N/A
Recruitment	Ten healthy plasma samples were purchased from Precision for Medicine. Out of 20 GBM plasma clinical samples that we tested, four GBM samples were purchased from Precision for Medicine and the remaining 16 samples were received from Andrew Scott and Hui Gan, Tumour Targeting Laboratory, ONJCRI, Melbourne, Australia.
Ethics oversight	University of Notre Dame

Note that full information on the approval of the study protocol must also be provided in the manuscript.

Field-specific reporting

Please select the one below that is the best fit for your research. If you are not sure, read the appropriate sections before making your selection.

☒ Life sciences ☐ Behavioural & social sciences ☐ Ecological, evolutionary & environmental sciences

For a reference copy of the document with all sections, see nature.com/documents/nr-reporting-summary-flat.pdf

Life sciences study design

All studies must disclose on these points even when the disclosure is negative.

Sample size	The biomarker need to be validated was active EGFR and we wanted its AUC 95% CI to be reasonably small. For our current sample size, it was within 0.99 which is reasonably good for a small pilot study involving active EGFR.
Data exclusions	No data was excluded.
Replication	Each experiments were replicated at least three times.
Randomization	No randomization was done for the calibration curves, and pure EV samples of known concentrations were used. No randomization was done for clinical samples as it was initial proof of concept/small pilot study.
Blinding	No blinding was performed as it was small cohort study.

Reporting for specific materials, systems and methods

We require information from authors about some types of materials, experimental systems and methods used in many studies. Here, indicate whether each material, system or method listed is relevant to your study. If you are not sure if a list item applies to your research, read the appropriate section before selecting a response.

Materials & experimental systems

n/a	Involved in the study	
☐	☒	Antibodies
☒	☐	Eukaryotic cell lines
☒	☐	Palaeontology and archaeology
☒	☐	Animals and other organisms
☐	☒	Clinical data
☒	☐	Dual use research of concern
☒	☐	Plants

Methods

n/a	Involved in the study	
☒	☐	ChIP-seq
☒	☐	Flow cytometry
☒	☐	MRI-based neuroimaging

Antibodies

Antibodies used	Anti-CD63 (BD Biosciences 556019, Clone H5C6, Lot# 9149755)

Antibodies used	Anti-EGFR (ABT-806)(Creative Biolabs TAB-228CL, Lot#CB2011R01)<br>Anti-EGFR (Cetuximab)(R&D Systems MAB9577-100, Clone #Hu1, Lot#CKIN0722031)
Validation	Done by the manufacturer.<br>Anti-CD63 (BD Biosciences 556019): https://www.bdbiosciences.com/en-eu/products/reagents/flow-cytometry-reagents/research-reagents/single-color-antibodies-ruo/purified-mouse-anti-human-cd63.556019<br>Anti-EGFR (ABT-806)(Creative Biolabs TAB-228CL): https://www.creativebiolabs.net/Anti-EGFR-Therapeutic-Antibody-ABT-806-19398.htm<br>Anti-EGFR (Cetuximab)(R&D Systems MAB9577-100): https://www.rndsystems.com/products/human-egfr-research-grade-cetuximab-biosimilar-antibody-hu1_mab9577

Clinical data

Policy information about clinical studies

All manuscripts should comply with the ICMJE guidelines for publication of clinical research and a completed CONSORT checklist must be included with all submissions.

Clinical trial registration	*Provide the trial registration number from ClinicalTrials.gov or an equivalent agency.*
Study protocol	*Note where the full trial protocol can be accessed OR if not available, explain why.*
Data collection	*Describe the settings and locales of data collection, noting the time periods of recruitment and data collection.*
Outcomes	*Describe how you pre-defined primary and secondary outcome measures and how you assessed these measures.*
